# Gut microbiota-derived acetate attenuates lung injury induced by influenza infection via protecting airway tight junctions

**DOI:** 10.1186/s12967-024-05376-4

**Published:** 2024-06-15

**Authors:** Lei Hu, Li Sun, Chun Yang, Da-Wei Zhang, Yuan-Yuan Wei, Ming-Ming Yang, Hui-Mei Wu, Guang-He Fei

**Affiliations:** 1 Department of Respiratory and Critical Care Medicine, First Affiliated Hospital of Anhui Medical University, Hefei, China; 2 Key Laboratory of Respiratory Diseases Research and Medical Transformation of Anhui Province, Hefei, China; 3 Department of Emergency Intensive Care Unit, First Affiliated Hospital of Anhui Medical University, Hefei, China; 4 Department of Geriatric Respiratory and Critical Care Medicine, Anhui Geriatric Institute, The First Affiliated Hospital of Anhui Medical University, Hefei, China

**Keywords:** Gut microbiota, Gut-lung axis, Acetate, Tight junctions, Lung injury, Influenza A virus

## Abstract

**Background:**

Gut microbiota (GM) have been implicated as important regulators of gastrointestinal symptom which is commonly occurred along with respiratory influenza A virus (IAV) infection, suggesting the involvement of the gut-to-lung axis in a host’s response to IAV. IAV primarily destroys airway epithelium tight junctions (TJs) and consequently causes acute respiratory disease syndrome. It is known that GM and their metabolism produce an anti-influenza effect, but their role in IAV-induced airway epithelial integrity remains unknown.

**Methods:**

A mouse model of IAV infection was established. GM were analyzed using 16S rRNA gene sequencing, and short-chain fatty acids (SCFAs) levels were measured. GM depletion and fecal microbiota transplantation (FMT) were conducted to validate the role of GM in IAV infection. A pair-feeding experiment was conducted to reveal whether IAV-induced GM dysbiosis is attributed to impaired food intake. Furthermore, human bronchial epithelial (HBE) cells were cocultured with IAV in the presence or absence of acetate. TJs function was analyzed by paracellular permeability and transepithelial electronic resistance (TEER). The mechanism of how acetate affects TJs integrity was evaluated in HBE cells transfected with G protein-coupled receptor 43 (GPR43) short hairpin RNA (shRNA).

**Results:**

IAV-infected mice exhibited lower relative abundance of acetate-producing bacteria (Bacteroides, Bifidobacterium, and Akkermansia) and decreased acetate levels in gut and serum. These changes were partly caused by a decrease in food consumption (due to anorexia). GM depletion exacerbated and FMT restored IAV-induced lung inflammatory injury. IAV infection suppressed expressions of TJs (occludin, ZO-1) leading to disrupted airway epithelial barrier function as evidenced by decreased TEER and increased permeability. Acetate pretreatment activated GPR43, partially restored IAV-induced airway epithelial barrier function, and reduced inflammatory cytokines levels (TNF-α, IL-6, and IL-1β). Such protective effects of acetate were absent in HBE cells transfected with GPR43 shRNA. Acetate and GPR43 improved TJs in an AMP-activated protein kinase (AMPK)-dependent manner.

**Conclusion:**

Collectively, our results demonstrated that GM protected airway TJs by modulating GPR43-AMPK signaling in IAV-induced lung injury. Therefore, improving GM dysbiosis may be a potential therapeutic target for patients with IAV infection.

**Supplementary Information:**

The online version contains supplementary material available at 10.1186/s12967-024-05376-4.

## Introduction

Despite widespread use of anti-influenza virus drug treatment and vaccination programs, influenza A virus (IAV) infection causes more than 300,000–500,000 deaths annually [1, 2]. Patients infected by IAV typically experience respiratory symptoms, frequently accompanied by gastrointestinal symptoms such as anorexia, abdominal pain and diarrhea, but the potential ties between lung and gut are unknown [3, 4]. Abnormalities in the gut microbiota (GM) composition have been implicated in a variety of pulmonary diseases, for example, pneumococcal pneumonia [5], chronic obstructive pulmonary disease [6], and allergic airway disease [7]. Considering the vital importance of the gut-lung axis in lung diseases, we hypothesize that dysbiosis of the GM during infection contributes to the pathogenesis of IAV– induced lung injury.

IAV infection disrupts the airway epithelial cell barrier causing acute respiratory disease syndrome and which can lead to death [8, 9]. As the first physical barrier, the epithelium serves as an essential component of the body’s immune system that forms a barrier against environmental particles and pathogens [10]. Defects in the epithelial barrier, the so-called leaky epithelium, leave the host susceptible to respiratory pathogens [11]. Tight junctions (TJs), inter-cellular junctions situated in the apical-most area of cell–cell contact, are essential for establishing epithelial polarity and maintaining the epithelial barrier [12, 13]. Transmembrane proteins, such as occludin, span the cell gap and restrict the paracellular zone [14]. The intracellular domains of transmembrane proteins interact with adapter proteins, for instance zonula occludens proteins (ZO-1, ZO-2, and ZO-3), which bind to the actin cytoskeleton to stabilize the junctional structure [14, 15]. Emerging evidence suggests that airway TJs are targets of IAV infection causing airway barrier dysfunction [10].

The gastrointestinal tract possesses the largest and most diverse microbiota [16]. Close interplay between the GM and the host is essential in maintaining homeostasis of the organism. The effects of GM on the host are not restricted to the intestinal tract but extend to remote organs, such as the lungs [17]. The bidirectional crosstalk between the gut and the lungs is termed the “gut-lung axis” [18–20]. The end products of the fermentative activity of the GM are the short-chain fatty acids (SCFAs) acetate, propionate, and butyrate [16]. In addition to their role as fuel for colonocytes, SCFAs participate in regulation of host innate immune responses [21] by combining with the G protein-coupled receptors (GPR41, GPR43), and acetate is the most potent activator of GPR43 [16]. There is evidence that SCFAs prevent lung injury from respiratory viral infection and therefore are prospective agents for the treatment of IAV infection [22]. Disturbances of GM have been associated with many chronic respiratory diseases, but few have been well studied. Although IAV infection disrupted epithelial TJs [10, 23], whether changing GM composition affected airway epithelial cell TJs via the gut-lung axis was not determined.

In this study, we established a mouse model of IAV infection. The IAV-infected group exhibited a lower relative abundance of acetate-producing bacteria. Acetate protected airway TJs by modulating GPR43-AMPK signaling in IAV-induced lung injury. Our results revealed the critical role of the microbial metabolite acetate in airway barrier functions by regulating the gut-lung axis.

## Materials and methods

### Human study design

Twenty-six subjects (13 IAV patients, 13 age-, gender-, and BMI-matched control subjects) were recruited between December 2022 and June 2023. IAV infections were confirmed by real-time reverse-transcription polymerase chain reaction. Participants were excluded if they had received treatment with an antibiotic or probiotics, had experienced significant abdominal pain, bloating, or diarrhea in the previous 4 weeks, or had a previous history of gastrointestinal disease and respiratory diseases [24, 25].

### Virus strains and cells

H3N2 virus (A/Anhui/1/2017) was isolated from the patient in 2017. We performed all experiments that involved the propagation and infection of viruses in a biosafety level 2 laboratory. The influenza A (H3N2) virus were amplified in chicken embryonic eggs [26] and Madin-Darby canine kidney (MDCK) cells, and virus titers were assayed by standard plaque assay on MDCK cells [27].

Human bronchial epithelial cells (HBE, CRL-2741) were cultured in RPMI 1640 culture medium which contained 10% fetal bovine serum (FBS) and 1% penicillin/streptomycin at 37 °C with 5% CO_2_. Cell passaging and subsequent experiments were performed when the HBE cells reached a confluence of 80%.

### Animal model

C57BL/6 J male mice (6–8 weeks old) were purchased from the Experimental Animal Center of Anhui Province (Hefei, China). Mice were housed in a 12-h light/dark cycle with free access to food and water. Mouse body weights were recorded daily.

To explore the impact of GM on IAV progression, mice were divided randomly into seven groups according to treatment: (1) MOCK group: 50 μl of sterile PBS infected intranasally; (2) H3N2 group: 100 PFU (50 μl in sterile PBS) infected intranasally; (3) ABX group: to construct a model of GM depletion in mice, a cocktail of antibiotics (ABX) (ampicillin 1 g/l, metronidazole 1 g/l, neomycin sulfate 1 g/l, and vancomycin 0.5 g/l) were added to the drinking water over a period of 3 weeks, as previously described [5, 28]; (4) ABX + MOCK group: 3 days after stopping antibiotic drinking water, ABX mice were given 50 μl of sterile PBS infected intranasally; (5) ABX + H3N2 group: 3 days after discontinuing antibiotic drinking water, ABX mice were infected with 100 PFU (50 μl in sterile PBS) intranasally; (6) FMT MOCK group (transplantation of GM from MOCK mice to ABX mice): the FMT experiments were based on described studies [5, 18, 29]. Feces from the donor group of MOCK mice were resuspended in PBS at 0.125 g/ml. 100 μl of fecal suspension was administered by gavage to ABX mice (recipient mice) on 3 consecutive days. (7) FMT H3N2 group (transplantation of GM from H3N2 mice to ABX mice): transplantation of H3N2 mice feces to ABX mice as described above.

To determine the effects of acetate on IAV infected mice, acetate (Cat# S2889 Sigma-Aldrich) in drinking water (100 or 200 mM) or vehicle was administered based on a previous study 1 week prior to H3N2 infection [30, 31].

### DNA extraction and 16S rRNA sequencing

Genomic DNA from intestinal flora was extracted with genomic DNA kit (M5636-02; Omega Bio-Tek). The V3V4 region of the bacterial 16S rRNA gene, which is highly variable and about 468 bp in length, was used for sequencing, and the forward primer 338F (5′-ACTCCTACGGGGAGGCAGCA-3′) and the reverse primer 806R (5′-GGACTACHVGGGTWTCTAAT-3′) were applied for PCR amplification. Next, a 2 × 250 bp paired-end sequence was analyzed on an Illumina MiSeq platform. The resulting reads were analyzed as described [32].

### Quantification SCFAs

SCFAs in cecal contents, feces, BALF, and serum were tested with gas chromatography/mass spectrometry (GC/MS) [33]. Gas chromatography measurements of all samples was conducted on a trace 1300 gas chromatograph. Mass spectrometry analysis of the samples was conducted on an ISQ 7000. Samples were homogenized with glass beads and water, and mixtures were centrifuged. The supernatant was homogenized by adding 15% phosphoric acid, 375 μg/mL 4-methylpentanoic acid solution, and ether. The sample supernatant obtained was analyzed by GC–MS [34, 35].

### Pair-feeding experiments

For food intake pairing experiments, the daily food consumption of mice in the IAV group was calculated. Five IAV-infected mice were placed in a single cage and the consumption of food was recorded every 24 h. The amount of food consumed was divided by 5 to obtain the amount of food consumed per day by the mice in the food pairing group. The pair-fed mice were restricted to 57% (day 3), 70% (day 4), 40% (day 5), 43% (day 6), and 47% (day 7) based on the food intake of the influenza group. The goal of food restriction was to achieve a 15% (7 dpi) body weight loss. Pair-fed animals were offered food twice daily and allowed water continually. Mice in the regular feeding (control) group had free access to food and water. Weight change of mice was measured daily, cecal contents and serum were collected after seven days.

### Bronchoalveolar lavage fluid (BALF)

Bronchoalveolar lavage fluid (BALF) was collected according to published methods [36]. Mice were tracheostomized under anesthesia with sodium pentobarbital. A nylon string was placed under the trachea, and a tiny hole was made in the front wall of the trachea through which the 22-gauge needle was inserted. The string was tied to the trachea. A total of 3.2 ml of cold PBS was injected slowly into the tiny hole in 4 separate injections and aspirated. Then the BALF obtained was centrifuged and the supernatant was used in subsequent experiments.

### Histopathology

Lung sections of 4 μm thickness were subsequently stained with hematoxylin and eosin. Mice sections were viewed microscopically in a blinded manner, and pathology findings were scored on a semiquantitative histologic score system [37, 38]. The degree of lung lesions was categorized into grades 0–4 for bronchiectasis, inflammatory cell infiltration, alveolitis, interstitial inflammation, hemorrhage, and edema.

### Real-time quantitative PCR analysis

The total RNA was extracted with TRIzol reagent (15596–018, Ambion). The RNA was immediately reverse transcribed into cDNA. The cDNA was used immediately for mRNA detection (11201ES08, Yeasen Biotech). Expression levels of all samples were standardized to ACTB levels in one sample, and relative expression was calculated through the 2^−△△Ct^ method.

### Western blot

HBE cells of 6-well plates were lysed with RIPA buffer (100 μl/10^6^ cells). Cell lysates were sonicated and incubated on ice (30 min) and centrifuged. The total proteins were separated by SDS-PAGE gel and transferred to a PVDF membrane. The membrane was blocked with 5% nonfat milk and incubated with primary antibodies β-actin (1:1000, ab227387, Abcam), ZO-1(1:1000, ZO1-1A12, Invitrogen), occludin (1:1000, ab216327, Abcam), GPR43 (1:200, sc-293202, Santa), AMPK (1:1000, 5832 Cell Signaling Technology) and p-AMPK (1:1000, 2535 Cell Signaling Technology) overnight at 4 °C. The membrane was then incubated with HRP-conjugated secondary antibody (1:3000) for 2 h and detected with enhanced chemiluminescence reagents (SQ201, Epizyme Biomedical Technology).

### Immunofluorescence staining

HBE cells were washed three times with cold PBS. Then cells were fixed with 4% paraformaldehyde and permeabilized with 0.25% Triton X-100. The cells were blocked with 5% bovine serum albumin and incubated with primary antibodies occludin (1:100, ab216327, Abcam) and ZO-1 (1:100, ZO1-1A12, Invitrogen) overnight at 4 °C. The cells were stained with fluorescent secondary antibodies anti-mouse IgG H&L (Alexa Fluor^®^ 594) and anti-rabbit IgG H&L (Alexa Fluor^®^ 488) for 1 h in the dark. Finally, cell nuclei were stained with 4',6-diamidino-2-phenylindole (DAPI) and fluorescent images were taken under a Zeiss fluorescence microscope.

### Transepithelial electrical resistance (TEER)

HBE cells (passage 3 or 5) were cultured in transwell chambers (Corning, 3460) and measured with an epithelial tissue voltohmmeter (EVOM2, World Precision Instruments, USA). The long-footed electrode piece of the voltohmmeter was kept in the lower chamber of the transwell, and the short-footed electrode piece was kept in the upper chamber of the transwell and suspended. TEER (Ωcm^2^) = (R_sample_—R_blank_) × effective membrane area (cm^2^) [39].

### Permeability assay

The calculated percentage leakage of fluorescein isothiocyanate (FITC)-dextran (46,944, Sigma-Aldrich) from apical to basolateral side was used to indicate epithelial permeability [40]. 1 mg/ml of FITC-dextran and culture medium were added to the apical and basal chambers, respectively, and the cultures were collected separately after 2 h of incubation in the dark. Fluorescence was measured with a fluorometer.

### Lentiviral infection

To knockdown GPR43, the shRNA sequence: 5′-GATCCGGCAATGAAATTACCTGCTACGAGACTCGAGTCTCGTAGCAGGTAATTTCATTGCCTTTTTTG-3′ was selected to construct the lentiviral vector (Hanbio, Shanghai, China). When the HBE cells in 6-well plates were at 30% confluence, they were treated with lentivirus (MOI 20) and polybrene (6 mg/ml), which were replaced with fresh medium after 24 h. Following three days of lentiviral infection, the HBE cells were collected and assayed for GPR43 expression by Western blot analysis.

### Enzyme-linked immunosorbent assay (ELISA)

Mouse serum and BAIF cytokine levels were determined with mouse TNF-α ELISA kit (E-EL-M3063, Elabscience), mouse IL-6 (E-EL-M0044c, Elabscience), and mouse IL-1β ELISA kit (E-EL-M0037c, Elabscience) according to the manufacturers’ instructions.

### Statistical analysis

All statistics were performed with GraphPad Prism 8.0.2. Continuous data were presented as mean ± standard deviation (SD) when normality tests were passed. The median (interquartile range; 25th–75th percentile) was used to describe variables for non-normal distributions. Two independent groups that met the normality and the variance were compared using the Student’s t–test. Otherwise, the Mann–Whitney U test was performed. Differences among multiple groups were performed with one-way ANOVA. Mouse survival was analyzed with Kaplan-Mill survival curves and the log-rank test (Mantel-Cox). *P* < 0.05 was considered statistically significant (****, *P* < 0.0001; ***, *P* < 0.001; **, *P* < 0.01; *, *P* < 0.05).

## Results

### Total SCFAs and acetate levels in feces and serum were lower in patients with IAV infection

The study subjects consisted of 13 patients with IAV infection and 13 healthy IAV-free subjects. No significant differences in baseline data (age, gender, and body mass index) were observed between the two populations (Table 1). In the IAV group, 76.92% of patients had fever on admission, with a median temperature of 38.20 °C (IQR, 37.05–38.75), and 23.08% of patients had diarrhea. Patients with IAV infection exhibited a significantly lower lymphocyte count compared to the control group. Fecal measurements of total SCFAs and their three main components were all lower in patients with IAV infection than in the control group. Serum measurements of total SCFAs and acetate concentrations were lower in patients with IAV infection than in the control group (*P* < 0.01), whereas the concentrations of propionate and butyrate in the IAV-infected group and control group were not significantly different (Table 1).

**Table 1:** Baseline characteristics of controls and patients with IAV

Variables	Controls (N = 13)	IAV (N = 13)	*P*-value
Age, year	57.85 ± 5.23	59.38 ± 8.08	0.57^a^
Female sex—no./total no. (%)	6/13 (0.46)	7/13 (0.54)	> 0.99^c^
BMI, kg/m^2^	22.43 ± 2.09	22.73 ± 2.85	0.76^a^
Symptoms—no. (%)			
Fever on admission	NA	10/13 (76.92)	NA
Temperature on admission (°C)	NA	38.20 (37.05–38.75)	NA
Cough	NA	11/13 (84.62)	NA
Diarrhea	NA	3/13 (23.08)	NA
White blood cell count, × 10^9^/L	5.37 ± 1.02	6.56 ± 1.83	0.05^a^
Neutrophil count, × 10^9^/L	3.12 ± 0.61	3.93 ± 1.18	0.04^a^
Lymphocyte count, × 10^9^/L	1.74 ± 0.50	1.27 ± 0.40	0.01^a^
Platelet count, × 10^9^/L	189.6 ± 41.20	202.5 ± 45.48	0.46^a^
Haemoglobin level, g/L	145.0 (129.0–152.5)	146.0 (138.5–158.5)	0.52^b^
C-reactive protein level ≥ 10 mg/L—no./total no. (%)	NA	9/13 (69.23)	NA
SCFAs concentration in feces (μg/g)			
Total SCFAs	629.5 ± 185.6	165.3 ± 121.8	< 0.0001^a^
Acetate	415.7 ± 127.9	99.94 ± 70.96	< 0.0001^a^
Propionate	142.9 ± 45.34	41.63 ± 71.37	0.0002^a^
Butyrate	70.81 ± 44.51	23.70 ± 31.04	0.0045^a^
SCFAs concentration in serum (μg/mL)			
Total SCFAs	4.70 ± 1.60	3.06 ± 0.52	0.0018^a^
Acetate	4.02 ± 1.44	2.48 ± 0.47	0.0012^a^
Propionate	0.58 ± 0.17	0.50 ± 0.11	0.18^a^
Butyrate	0.09 (0.07–0.12)	0.08 (0.06–0.10)	0.48^b^

Data are presented as number (%) or means ± standard deviation or median (interquartile range)

*IAV* influenza A virus, *BMI* body mass index, *SCFAs* short-chain fatty acids, *NA* not available

^a^t-test

^b^Mann-Whitney U test

^c^χ^2^ test. P < 0.05 was considered as statistically significant

### Influenza infection changed the composition and metabolic output of the GM

**Fig. 1:**
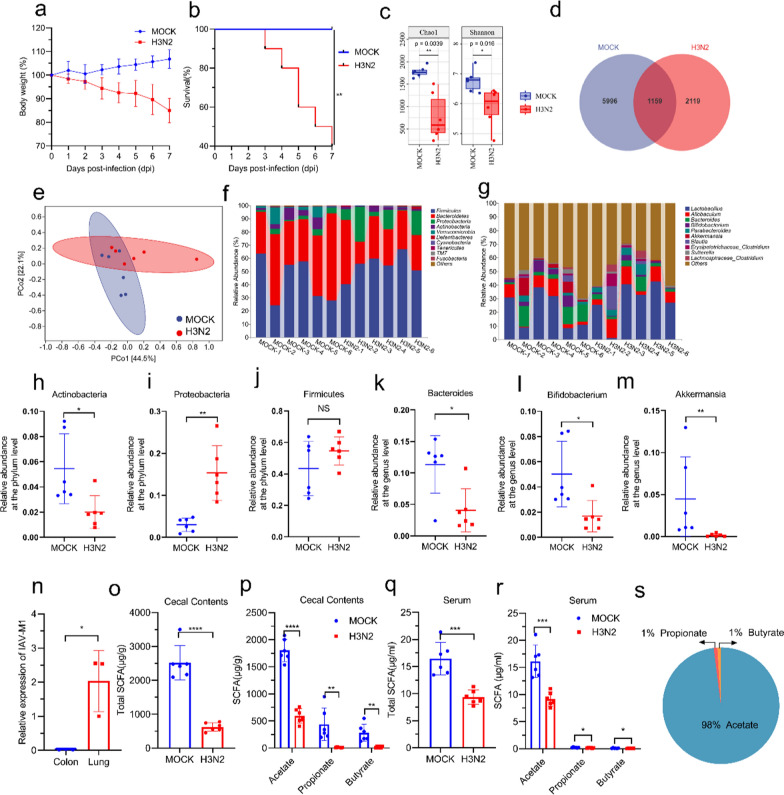
GM composition and levels of SCFAs change during IAV infection. **a**, **b** Mice were infected with H3N2. Body weight changes and survival rates were recorded. **c** Comparison of indexes of alpha diversity (Chao1 and Shannon index) between MOCK and H3N2 groups. **d** Venn diagram representation of amplicon sequence variation (ASV) between MOCK and H3N2 groups. **e** β diversity index of PCoA with weighted_Unifrac distance. **f**, **g** Relative abundance of GM in the MOCK and H3N2 mice (at the phylum and genus levels, respectively). **h**–**j** The relative abundance of Actinobacteria, Proteobacteria and Firmicutes between two groups were analyzed. **k**–**m** The relative abundance of Bacteroides, Bifidobacterium and Akkermansia between two groups were analyzed. **n** The levels of IAV M1 mRNA in mouse lungs and colon were measured by quantitative RT-PCR. **o**–**r** Concentrations of total SCFAs and individual concentrations of acetate, propionate, and butyrate in mice cecum and serum. **s** Major components of serum SCFAs in MOCK mice. Data are expressed as mean ± SD. *P < 0.05, **P < 0.01, ***P < 0.001, ****P < 0.0001

To investigate the effects of IAV (H3N2 strain) infection on the mouse GM composition, we compared the GM between MOCK and IAV-infected mice. H3N2-infected mice showed significantly body weight loss and increased mortality (up to 60%) (Fig. 1a, b). Alpha diversity is an indicator of species richness, diversity and evenness in a locally homogeneous habitat, usually assessed by Chao1 index (for community diversity detection) and Shannon index (for total species estimation) [41, 42]. Consistently, alpha diversity was significantly lower in H3N2-infected mice as compared to that in the MOCK mice (Fig. 1c). The Venn diagram (Fig. 1d) displayed 5996 and 2119 amplicon sequence variants (ASVs) in the MOCK and H3N2 groups, respectively, with 1159 ASVs shared between the two groups. The principal coordinate analysis (PCoA) showed that GM in the H3N2-infected mice were different from GM in the MOCK mice (Fig. 1e). At the phylum level, the results in Fig. 1f, illustrated that H3N2 infection significantly reduced the relative abundance of Actinobacteria (Fig. 1h), while increased in the relative abundance of Proteobacteria (Fig. 1i). There was no difference in Firmicutes (Fig. 1j, P = 0.3939). At the genus level, the H3N2-infected group had lower relative abundance of Bacteroides, Bifidobacterium and Akkermansia (Fig. 1k–m). Linear discriminant analysis Effect Size (LEfSe) analysis was used to identify the most discriminative bacterial taxa between two groups. Bacteroides, Bifidobacterium and Akkermansia were identified to be preferentially more abundant in MOCK group (Figure S1a). Spearman correlation analysis of GM and SCFAs (Figure S1b) showed that Bacteroides, Bifidobacterium and Akkermansia were correlated positively with acetate. These results suggested that the decrease of acetate in the H3N2 group was related to lower relative abundance of acetate-producing bacteria Bacteroides, Bifidobacterium, and Akkermansia [16]. To exclude the possibility that IAV entered the gastrointestinal tract and subsequently affected GM, we examined the intestinal tissue for the presence of IAV. Results indicated that the intestine was free of IAV replication (Fig. 1n).

Our aforementioned clinical data suggested that SCFAs, especially acetate, are reduced in IAV-infected patients. Therefore, we next explored the content of SCFAs. The overall concentration of SCFAs in the cecum was significantly lower in H3N2 mice than in the MOCK mice, and the concentrations of acetate (the major component of SCFAs), propionate, and butyrate were all lower (Fig. 1o, p). As shown in Fig. 1q, r, IAV infection caused a decrease in the SCFAs concentration in the serum and acetate was the main SCFAs found in serum (Fig. 1s). Circulating acetate was significantly lower after influenza infection. Furthermore, acetate in BALF were significantly lower in H3N2 mice than in the MOCK mice (Figure S1c-d)**.** Our present results confirmed that acetate was a major component of circulating SCFAs, and its levels decreased significantly after IAV infection.

### GM depletion exacerbated influenza virus-induced lung inflammatory injury and reduced acetate level

**Fig. 2:**
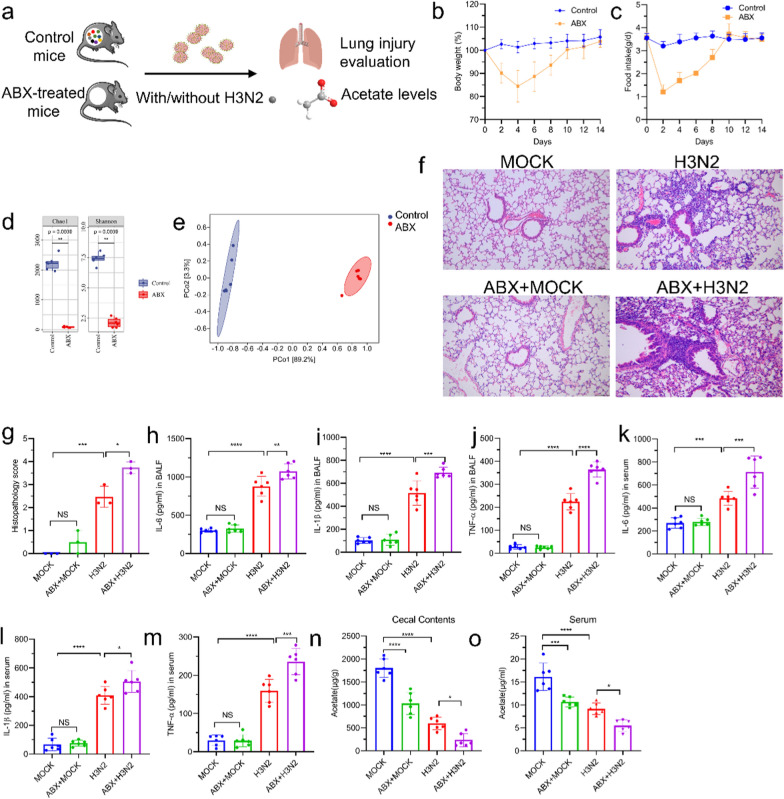
Influenza-induced lung injury and acetate level reduction were exacerbated by depletion of the GM. **a** Control mice and ABX mice were underwent MOCK or H3N2 infection, and lung injury severity and acetate concentrations were evaluated. **b**, **c** Body weight change and food consumption of ABX group and control group. **d** Comparison of indexes of alpha diversity (Chao1 and Shannon index) between control and ABX groups. **e** β diversity index of PCoA with weighted_Unifrac distance. **f**, **g** Representative H&E stained sections of lungs and histopathology scores (bar = 100 μm). **h**–**m** ABX treatment increased inflammatory cytokines (TNF-α, IL-1β, and IL-6) in BALF and serum. **n**, **o** ABX treatment resulted in decresed acetate concentrations in cecal contents and serum. Data are expressed as mean ± SD. *P < 0.05, **P < 0.01, ***P < 0.001, ****P < 0.0001

To determine the importance of GM composition in IAV-induced lung injury, we established the GM depletion mouse model by administering a broad-spectrum antibiotic (ABX) orally (Fig. 2a). After 10 days of adaptation, the ABX mice and the control mice exhibited no differences in body weight or food intake (Fig. 2b, c). In the ABX-treated group, compared to the control group, the alpha diversity was sharply reduced and the composition was significantly different (Fig. 2d, e). These data provide strong evidence of our success in establishing a mouse model of GM depletion. Mice with depleted GM showed robust lung injury after H3N2 infection as evidenced by increased histopathology scores (Fig. 2f, g) as well as levels of cytokines (TNF-α, IL-6, and IL-1β) in BALF (Fig. 2h–j) and serum (Fig. 2k–m). Acetate levels in cecum contents and serum were significantly decreased in ABX group mice after H3N2 infection (Fig. 2n, o). These results suggested that lower concentrations of acetate in the circulation were caused by changes in the GM. Inflammatory damage was more extensive in the lungs of mice with depleted GM during influenza.

### GM composition had a causal effect on IAV-induced lung injury likely via acetate

**Fig. 3:**
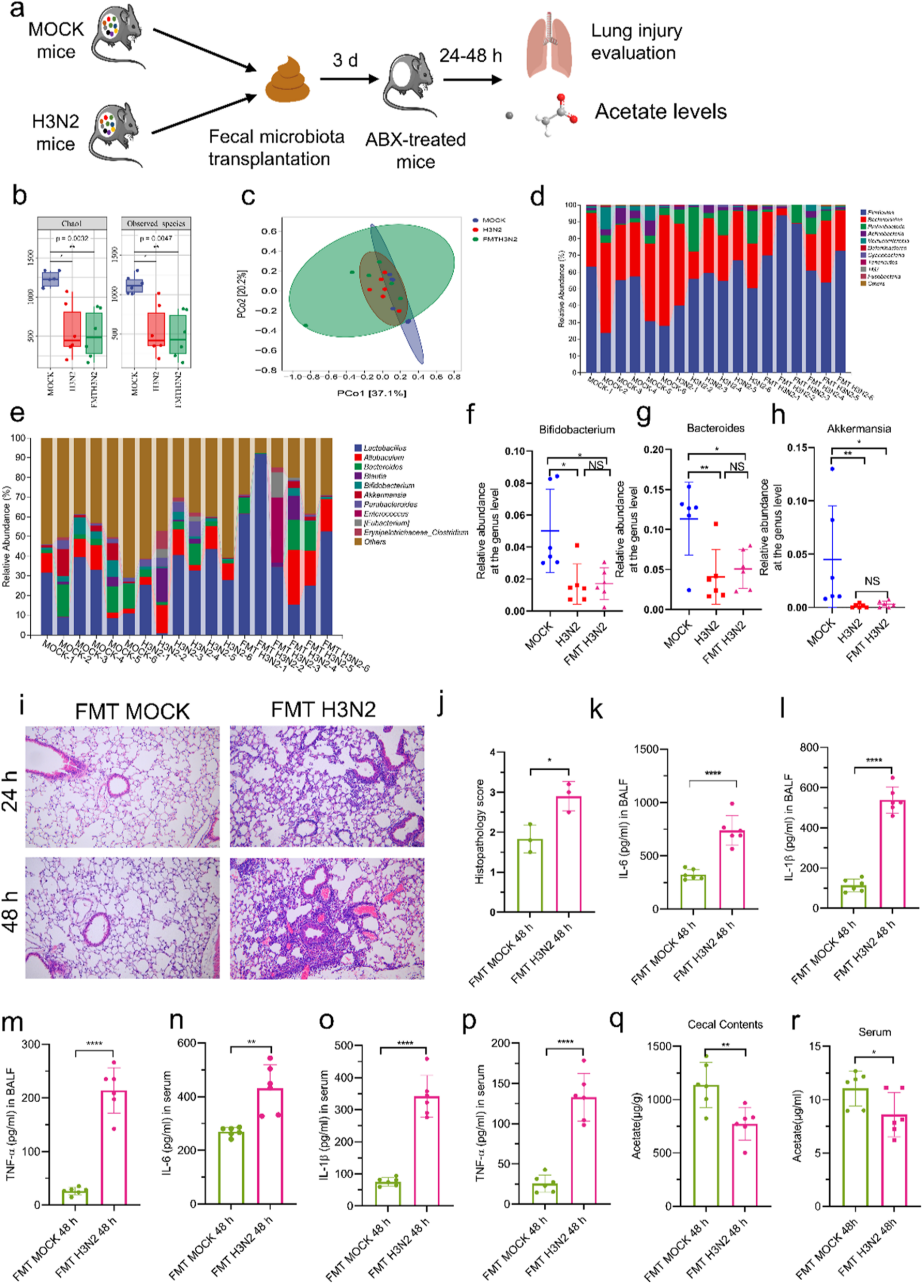
Transplantation of fresh fecal microbiota from influenza-infected mice into ABX mice led to lung injury and decreased acetate concentrations. **a** Transplantation of fresh feces collected from MOCK and H3N2 mice to ABX mice. **b** Comparison of indexes of alpha diversity among the three groups. **c** β diversity index of PCoA with weighted_Unifrac distance. **d** Microbial composition at the phylum level. **e**–**h** Microbial composition at the genus level. The relative abundance of Bacteroides, Bifidobacterium and Akkermansia in three groups were analyzed. **i**, **j** Representative images of H&E stained lung sections from FMT MOCK and FMT H3N2 groups (bar = 100 μm) and histopathological scores. **k**–**p** Levels of inflammatory cytokines (TNF-α, IL-1β, and IL-6) in BALF and serum after post-transplantation treatment of ABX recipient. **q**, **r** Acetate concentrations in cecum and serum of two transplant groups. Data are expressed as mean ± SD. *P < 0.05, **P < 0.01, ***P < 0.001, ****P < 0.0001

To test whether alterations in GM may have a causal effect on the pathogenesis of IAV-induced lung injury, we performed mice FMT experiments (Fig. 3a). Feces were collected from H3N2-infected mice (donors) and transplanted into ABX mice (recipients). The recipient mice lungs were obtained at 24 h and 48 h after 3 days of consecutive FMT (Fig. 3a). We assessed the GM of the recipient group (48 h) after FMT experiments. As showed in Fig. 3b, alpha diversity was significantly lower in FMT H3N2 mice than in MOCK mice, and there was no difference with H3N2 mice. Furthermore, the PCoA results showed that both FMT H3N2 and H3N2 mice had similar GM structures, which were clearly different from that of the MOCK mice (Fig. 3c). At the phylum level, compared to the MOCK mice, we observed that H3N2 and FMT H3N2 mice had increased relative abundance of Proteobacteria and decreased relative abundance of Actinobacteria (Fig. 3d and Figure S1e-f). No significant differences in the phylum Firmicutes were observed among the three groups (Figure S1g). At the genus level, the relative abundances of Bacteroides, Bifidobacterium and Akkermansia reduced in H3N2 mice, and these alteration were replicated in recipient mice after FMT administration (Fig. 3e–h). Higher histopathology scores (Fig. 3i, j) and cytokines level (Fig. 3k–p) in the lungs and serum were recorded in ABX mice receiving feces from H3N2 mice as compared to mice receiving feces from MOCK mice. Especially, ABX mice transplanted with H3N2 feces exhibited lower levels of acetate in cecum and serum compared to the MOCK group (Fig. 3q, r). FMT experiments confirmed that lung inflammatory injury induced by H3N2 infection can be transmitted through the GM, which may be closely related to GM metabolites such as acetate. The above results suggest that GM composition has a causal effect on IAV-induced lung injury and acetate may be an essential mediator of gut-lung axis.

### Acetate alleviated damage of airway epithelium TJs in IAV-infected mice

**Fig. 4:**
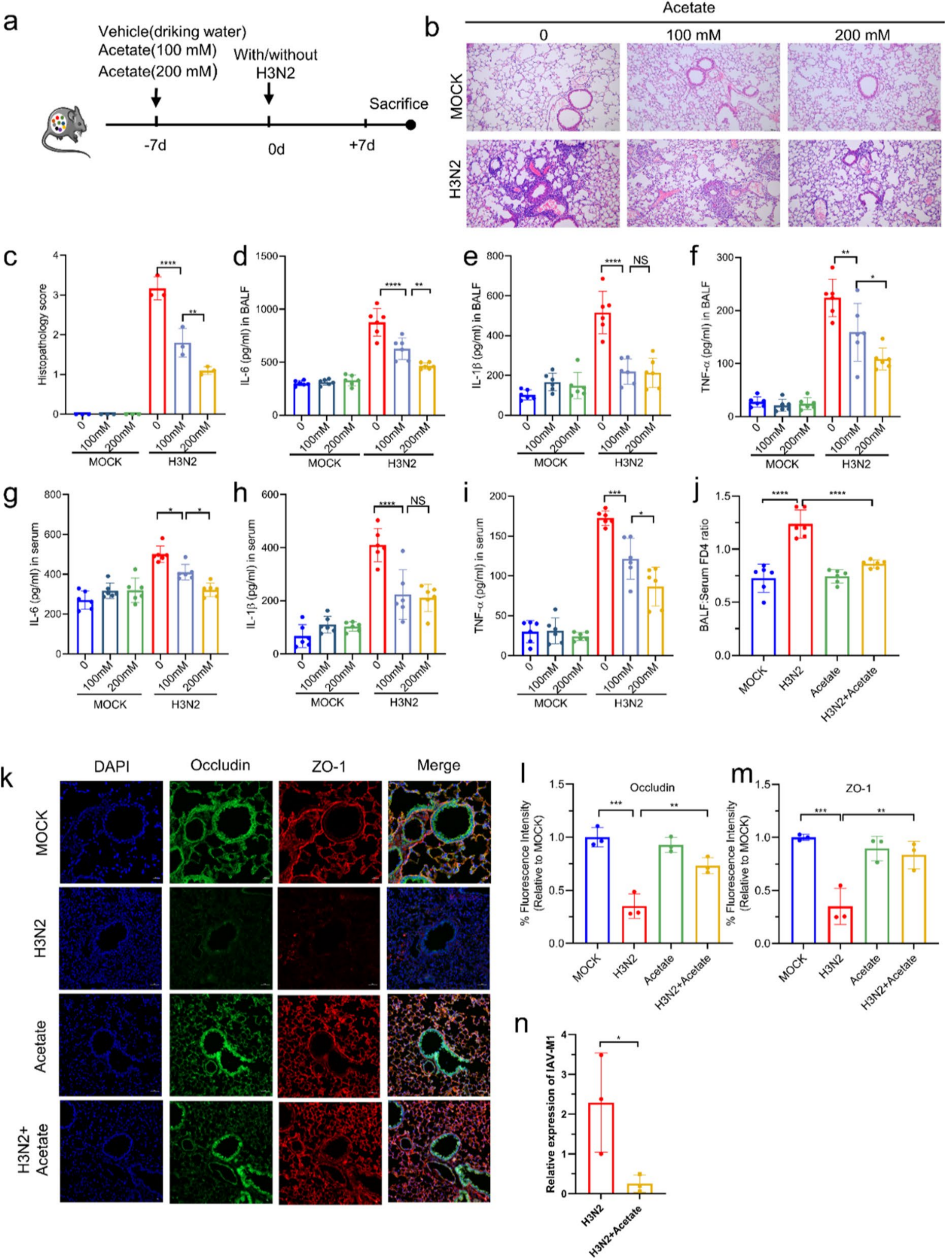
Acetate protected TJs and alleviated lung injury after H3N2 infection. **a** Mice received acetate (100 or 200 mM) in drinking water for 7 consecutive days before H3N2 infection. **b**, **c** H&E staining of representative mouse lung tissues after various concentrations of acetate intervention (bar = 100 μm) and histopathological scores. **d**–**i** Levels of inflammatory cytokines (TNF-α, IL-1β, and IL-6) in BALF and serum after acetate treatment. **j** Mice underwent FD4 injection into the tail vein, and the ratio of BALF to serum FD4 was determined. **k**–**m** Immunofluorescence staining of mice lung sections for occludin and ZO-1. **n** The levels of IAV M1 mRNA in mouse lungs were measured by quantitative RT-PCR. Data are expressed as mean ± SD. *P < 0.05, **P < 0.01, ***P < 0.001, ****P < 0.0001

We next investigated whether acetate by itself could protect against IAV infection. We treated mice with acetate (100 and 200 mM in drinking water) or vehicle prior to H3N2 infection (Fig. 4a). Inflammatory damage in the lungs was assessed according to histological scores (Fig. 4b, c) and inflammatory cytokine concentrations in the lungs (Fig. 4d–f) and serum (Fig. 4g–i). Both the severity of lung injury and cytokine concentrations were reduced in the acetate-treated group compared to the vehicle group, and these protective effects were dependent on the dose of acetate. We next evaluated the mechanism by which acetate ameliorates lung injury in H3N2 mice. Virus-induced alterations in epithelial TJ function reportedly leads to increased lung injury, so we first examined the airway barrier. Lung permeability was assessed by testing of lungs FD4 leakage after H3N2 infection with or without acetate intervention. The FD4 leakage (the ratio of BALF to serum FD4) was 0.73 in MOCK mice which was increased to 1.24 in H3N2 mice (*P* < 0.0001). Following acetate treatment, this ratio decreased to 0.86 (*P* < 0.0001), indicating that acetate was effective in protecting bronchial epithelial cell permeability from H3N2 (Fig. 4j). Moreover, acetate treatment protected the expression and structure of TJs proteins such as occludin and ZO-1 from IAV disruption (Fig. 4k–m). Acetate therapy significantly decreased the virus loads in the lung tissue (Fig. 4n). These results suggested that acetate alleviated damage of airway epithelium TJs in H3N2 mice.

### Reduced food intake was partially responsible for GM changes in IAV-infected mice

**Fig. 5:**
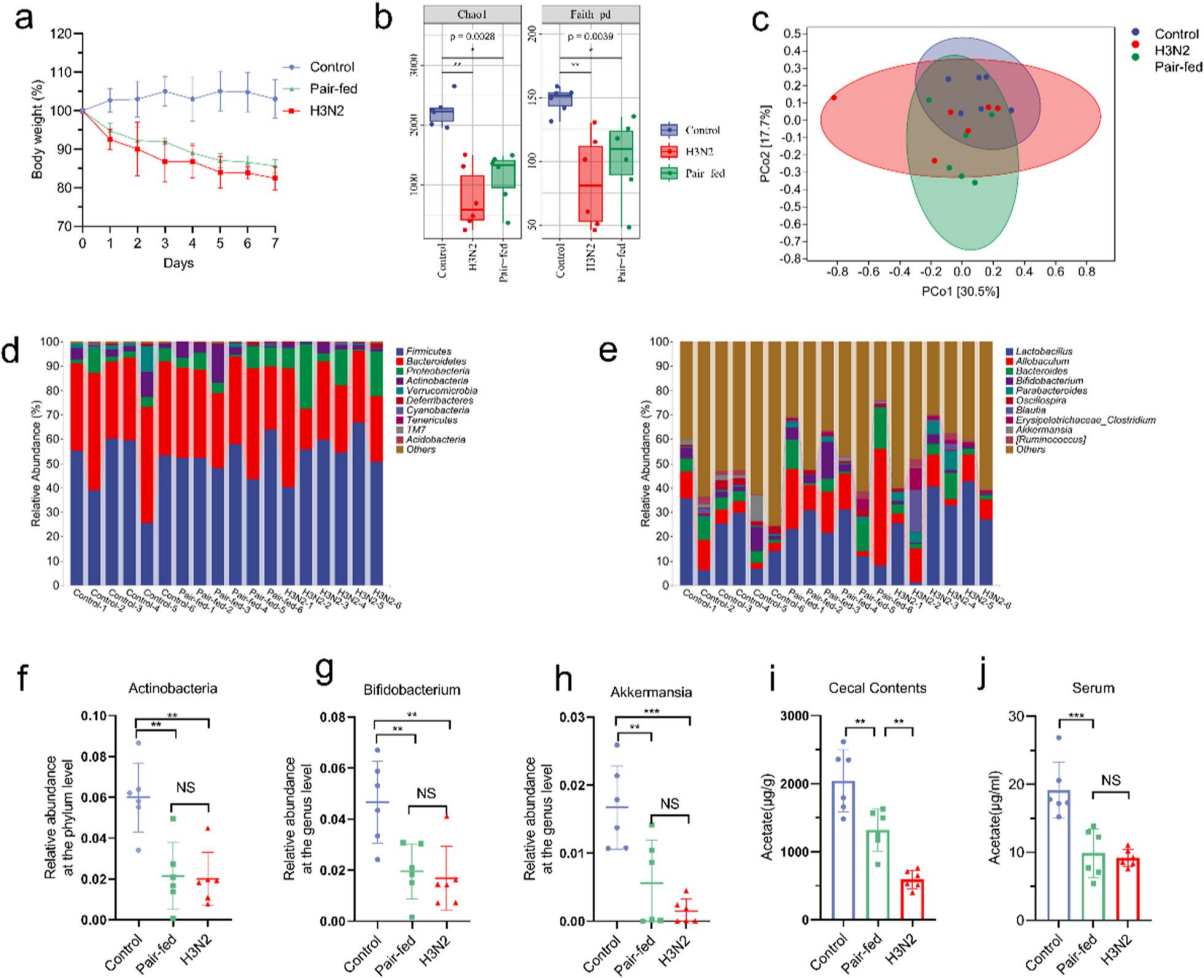
Altered GM in food restricted mice. **a** Changes in body weight were measured in control, pair-fed, and H3N2 group. **b** Comparison of indexes of alpha diversity (Chao1 and Faith_pd index) among control, pair-fed, and H3N2 groups. **c** PCoA with weighted_Unifrac distance of β diversity index. **d**, **e** Relative abundance of GM in the control, pair-fed, and H3N2 groups (at the phylum and genus levels, respectively). **f**–**h** The relative abundances of Actinobacteria, Bifidobacterium and Akkermansia in three groups were analyzed. **i**, **j** Cecum and serum acetate concentrations in control, pair-fed, and H3N2 groups. Data are expressed as mean ± SD. *P < 0.05, **P < 0.01, ***P < 0.001, ****P < 0.0001

The next question we asked was how IAV infection alters the composition of GM. Diet drives the composition and metabolism of the GM [16] and rapidly decreasing food consumption can change the GM composition and metabolites [43]. Thus, we investigated the relationship between reduced food intake (due to anorexia) and GM composition after IAV infection. In the H3N2 infection model, mice suffered food intake decrease and weight loss from the first day of infection, with a nadir on day 7 (causing weight loss of about 15–20%). We thus conducted a paired feeding experiment based on measuring food consumption of IAV-infected mice. As depicted in Fig. 5a, the pattern of weight loss in pair-fed mice was the same as in mice infected with IAV. Pair-fed mice had lost about 15% of their initial body weight at the time of sacrifice. Alpha diversity was significantiy lower in pair-fed mice than in control mice, but there was no difference with the H3N2 group (Fig. 5b). According to the analysis of β-diversity, the GM composition of the pair-fed mice was different from that of the control mice, while there was no difference with the H3N2 mice (Fig. 5c). At the phylum level, the GM of pair-fed mice had a significantly lower relative abundance of Actinobacteria than at baseline (Fig. 5d, f). At the genus level, the major change was a decrease in the abundance of Bifidobacterium and Akkermansia (Fig. 5e, g–h). Restriction of food intake did not completely replicate the GM composition of H3N2 mice, but it resulted in GM composition similar to that of the H3N2 mice. The levels of acetate in the cecum and circulation were lower in pair-fed mice compared with control mice (Fig. 5i, j). Therefore, food restriction that mimics influenza disease shifts the composition of GM in mice and reduces acetate levels in cecum and serum. We hypothesized that the influenza-induced change in GM composition was partly caused by the reduction in food intake.

### Acetate protected the TJs of bronchial epithelial cells from H3N2-induced damage

**Fig. 6:**
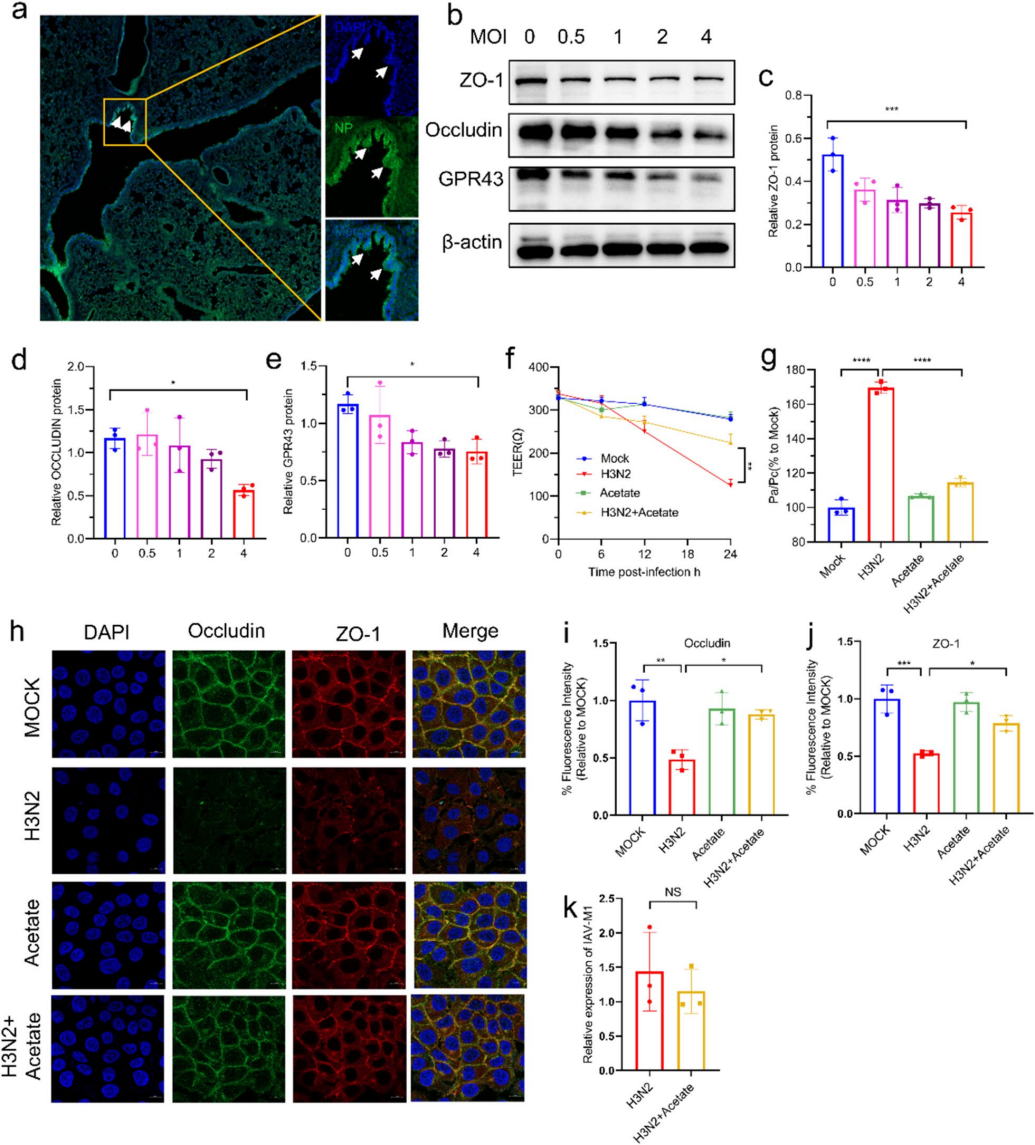
Roles of acetate in regulating epithelial TJs protein expression. **a** Immunofluorescence staining for viral NP in lung tissue of mice infected with influenza. **b**–**e** Analysis of occludin, ZO-1 and GPR43 protein expression by western blotting in HBE Cells after H3N2 infection. The expression of occludin, ZO-1, and GPR43 protein were quantified by Image J. **f** HBE cells were seeded into transwell chambers and TEER was measured at specified times after H3N2 infection in the presence or absence of acetate. **g** Permeability of HBE cell monolayers to FD4 measured after the indicated treatments. **h**–**j** Immunofluorescence staining for occludin and ZO-1 in HBE cells infected with H3N2. **k** The levels of IAV M1 mRNA in the HBE cells were measured by quantitative RT-PCR.Data are expressed as mean ± SD. *P < 0.05, **P < 0.01, ***P < 0.001, ****P < 0.0001

In mouse lung tissue sections, IAV replication was predominantly located in the airway epithelium (Fig. 6a). Therefore, we chose the airway epithelial cell line as the vector cells for in vitro research. As illustrated in Fig. 6b–e, there was a marked decrease in TJs protein at MOI = 4 after 24 h of infection. Next, we investigated whether acetate could attenuate the destruction of TJs. We treated the cells with various concentrations of acetate after influenza infection. As shown in Figure S2c-e, acetate showed the strongest protective effect at a concentration of 10 mM. Acetate intervention did not affect TEER values in non-infected cells. However, it prevented the disruption of barrier function (based on declining TEER values and increased epithelial permeability) caused by IAV infection (Fig. 6f, g). Immunofluorescence staining showed that acetate partially restored occludin and ZO-1 protein expression on the membranes of H3N2-infected HBE cells (Fig. 6h–j). However, acetate did not reduce viral titers in epithelial cells (Fig. 6k). The above results indicated that acetate protected the TJs of bronchial epithelial cells from H3N2-induced damage.

### The GPR43-AMPK pathway participated in the protection of TJs.

**Fig. 7:**
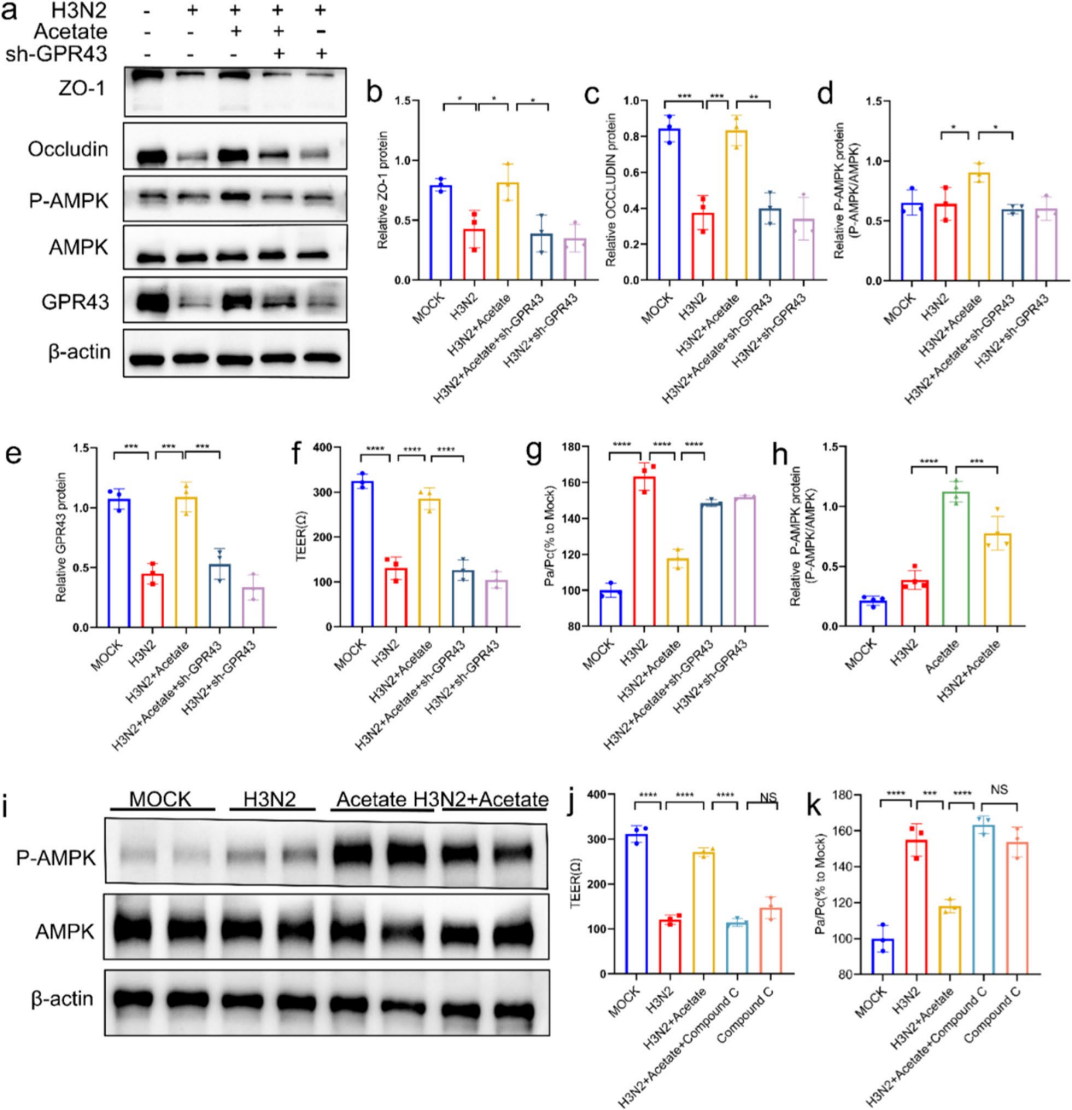
GPR43 mediated the protective effect of acetate on TJs of HBE cells. HBE cells were knocked down with or without shGPR43, and the cells were infected with H3N2 (MOI 4) for 24 h or not infected in the presence or absence of acetate (10 mM). **a**–**e** Analysis of ZO-1, occludin, GPR43, AMPK and P-AMPK protein expression in HBE cells by western blotting. **f** HBE cells were cultured in transwell chambers and TEER was measured. **g** Analysis of permeability in HBE cells. **h**, **i** Representative blots of AMPK and P-AMPK in mice lung tissue. **j**, **k** TEER and permeability of HBE cells were determined after cells were pretreated with Compound C (40 μM), H3N2 infected or uninfected, in the presence or absence of acetate (10 mM). Data are expressed as mean ± SD. *P < 0.05, **P < 0.01, ***P < 0.001, ****P < 0.0001

**Fig. 8:**
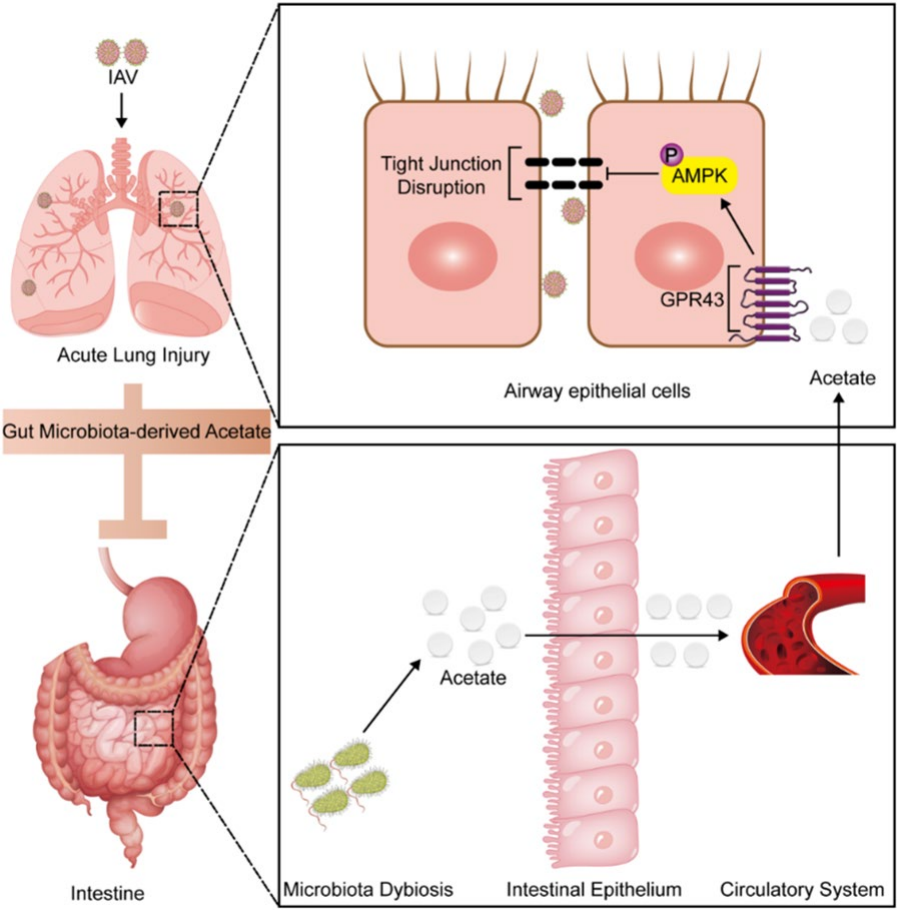
Gut microbiota-derived acetate is a key mediator of the gut-lung axis in lung injury induced by IAV infection. Schematic diagram illustrating IAV infection induced dysbiosis of GM, leading to a decrease in circulating acetate levels through the gut–lung axis. Improvement of GM, protection of airway epithelial TJs via GPR43-AMPK-dependent pathway and consequently reduced inflammatory lung damage

Finally, we investigated the molecular mechanism underlying the protective effect of acetate on TJs. AMP-activated protein kinase (AMPK) signaling is essential for TJs integrity [44] and polarization [45], and GPR43 has multiple beneficial effects on lipid and glucose metabolism in humans by regulating AMPK [46]. Thus, we hypothesized GPR43-AMPK may be mediated the protective effect of acetate on TJs. As shown in Fig. 7a–e, the protective effect of acetate was attenuated in HBE cells transfected with GPR43-shRNA. Acetate pretreatment failed to increase TEER and decrease epithelial permeability in HBE cells transfected with GPR43-shRNA (Fig. 7f, g). Acetate treatment induced phosphorylation of AMPK, but this upregulation was attenuated in GPR43 knockdown cells (Fig. 7d). We evaluated key proteins of AMPK signaling in lung tissue and found that acetate induced phosphorylation of AMPK in vivo (Fig. 7h, i). Furthermore, Fig. 7j, k showed that acetate failed to restore epithelial function when cells were treated with a specific pharmacological inhibitor of AMPK (compound C). Thus, the above data suggested that acetate protected TJ through the GPR43-AMPK pathway (Fig. 8).

## Discussion

Extensive research has shown that aberrations in the GM contribute to pulmonary diseases such as COPD [6], asthma [7], and pulmonary arterial hypertension [47]. The present study is the first time to determine the impact of GM-derived acetate on reducing inflammatory lung injury by protecting airway TJs from IAV infection. Our findings suggested that infection with IAV changed the composition of GM. Consequently, the metabolite acetate, the major component of SCFAs, was significantly reduced in the circulation. Acetate concentrations in the cecum and serum of Abx treated mice were reduced after infection with H3N2, implying that the GM is a source of acetate. The protective effect of acetate was attenuated in HBE cells after shRNA-induced knockdown of GPR43. The involvement of GPR43-AMPK signaling was validated both in vivo and in vitro. These data provide evidence that GM-derived acetate maintains the integrity of TJs and attenuates lung inflammatory injury after H3N2 infection, and that the GPR43-AMPK signaling pathway is involved in this process.

Recent studies have discussed the influences of IAV infection on the GM. The mouse model of IAV infection in the present study revealed a reduction in the community richness of microorganisms. Consistent with our findings, Gu [25] found that the diversity of the GM was lower in patients with H1N1 infection. Therefore, we concluded that the reduction in intestinal bacterial content is a generalized outcome of IAV infection, independent of the viral subtype. In the present study, IAV infection led to to a decrease in the abundance of several beneficial bacteria, including Bacteroides, Bifidobacterium, and Akkermansia, all of which have been shown to protect against IAV infection [3, 48, 49]. We speculated that supplementation with these beneficial bacteria may combat respiratory infections caused not only by the IAV but also by other respiratory viruses such as severe acute respiratory syndrome coronavirus 2 (SARS-CoV-2).

GM produce acetate, which is protective against IAV infection. However, it is noteworthy that in vivo experiments, lung virus titers decreased in mice pretreated with acetate, which had an antiviral effect (Fig. 4n). In contrast, in vitro experiments, acetate had been treated and did not have the ability to clear the virus (Fig. 6k). These results suggested that acetate acts on other immune cells to clear the virus in addition to protecting epithelial cell TJs. Sencio [30] showed that acetate promoted phagocytosis of macrophages to clear IAV. Similarly, although the protective effect of GM against IAV infection may be due to a variety of metabolites, acetate is probably the most important one.

Acetate has been reported to enhance host antiviral response [50], facilitate improvement of cognitive function [51], and modulate lung injury [52]. Our study revealed that acetate increased the expression of airway epithelial TJs, consequently alleviating barrier dysfunction in IAV-infected mice. Consistent with our findings, Fukuda [53] showed that acetate can also maintain defense functions of intestinal epithelial cells by inducing anti-inflammatory and/or anti-apoptotic effects. The basic element of the airway epithelium barrier is the intercellular physical structure, namely, the TJs [10, 11, 15]. Several studies have reported that promoting TJ formation improves the prognosis of IAV infection [8, 9, 14, 38]. Therefore, when acetate was protective in influenza-infected mice, we first considered epithelial TJs. Our work offered a new perspective: acetate attenuates IAV-induced inflammatory lung injury by maintaining airway epithelial TJ integrity.

In this study, acetate activated the downstream pathway by binding to its receptor GPR43. GPR43 has an essential function in the organization's metabolism and inflammation [54]. Our findings showed that acetate binds to the GPR43 receptor to reduce inflammation. Increasingly, studies have found that GPR43 regulates inflammatory responses differently in various settings. In a mouse model of gout, GPR43 was an essential requirement for activation of inflammasomes [55]. However, in another study [56], GPR43 inhibited the activation of inflammasomes by decreasing [Ca^2+^]_i_. This difference may be related to different disease backgrounds, cell types, and dosing concentrations. On the other hand, GPR43 has multiple beneficial effects on lipid and glucose metabolism in humans by regulating AMPK [46]. AMPK is an important sensor of cytoenergy and an adjuster of metabolic energy homeostasis at the whole-body level [57]. Compared with studies on the role of AMPK in metabolism, its role in innate immune regulation has been less well investigated. In our study, GPR43 attenuated the inflammatory response by regulating the AMPK pathway to maintain epithelial barrier function both in vivo and in vitro models. Recent findings have emphasized the importance of AMPK in regulating the assembly of epithelial TJs and maintaining epithelial barrier function [44, 58] rather than its function as a metabolic sensor. Activation of AMPKα/P53 signaling can inhibit influenza-induced inflammatory lung injury [59]. Consistent with our findings, Olivier [60] identified the role of AMPK in modulating the repair of mucosa and the regeneration of epithelium after colonic injury. The role of the AMPK pathway in immunity and inflammation requires further exploration.

In the present study, the GM metabolite acetate exerted anti-inflammatory effects through the GPR43-AMPK pathway. Several studies have proposed a regulatory role of GM and metabolites on type I interferons (IFNs) [50, 61]. The GM not only influences the acquired immune response, but it also affects adaptive immunity. Ichinohe [62] showed that the composition of the GM modulates the production of IAV-specific T cells (CD4 and CD8) and the antibody response after IAV infection. However, more research is warranted to provide an accurate understanding of how GM dysregulation affects inflammation both locally and systemically. There are several limitations in our study. First, it should be noted that although the 16S rRNA gene sequencing is widely used for microbiota characterization, it is limited in its ability to reveal genetic contents compared to metagenomic sequencing [63]. Second, it remains unclear the impact of IAV infection on SCFAs in human BALF sample. In addition, because of the multiple characteristics of the FMT and GPR43-AMPK pathways, more studies are needed to explore the pathways that mediate IAV progression after GM disorder.

## Conclusion

This work found that the GM metabolite acetate protected barrier function via the GPR43-AMPK pathway during IAV progression. Our findings suggested that bidirectional crosstalk between the gut and the lungs is critical in the fight against IAV infection. Furthermore, this work identified acetate, a microbiota metabolite, as a potentially important agent for the treatment of acute lung injury due to IAV infection in critically ill patients. Since there are limited preventive and therapeutic measures available for respiratory viral infections such as IAV infections and SARS-CoV-2 infection, the latter of which causes COVID-19, our findings may be helpful in designing strategies for interventions against respiratory viral infections in the future.

### Supplementary Information

Supplementary Material 1.

## Data Availability

The raw data of 16S rRNA sequencing generated in this study has been deposited under NCBI SRA BioProject, numbers PRJNA 1029756 (https://www.ncbi.nlm.nih.gov/sra/PRJNA1029756).

## Abbreviations

GM: Gut microbiota
IAV: Influenza A virus
TJs: Tight junctions
SCFAs: Short-chain fatty acids
FMT: Fecal microbiota transplantation
HBE: Human bronchial epithelial
TEER: Transepithelial electronic resistance
GPR43: G protein-coupled receptor 43
AMPK: AMP-activated protein kinase
BALF: Bronchoalveolar lavage fluid
