# Supplementary material for: Gut microbiota-derived acetate attenuates lung injury induced by influenza infection via protecting airway tight junctions
Source: J Transl Med. 2024 Jun 15;22:570. doi: 10.1186/s12967-024-05376-4 (PMC11179378; doi:10.1186/s12967-024-05376-4)
Supplement: Supplementary file 1 — Supplementary Material 1. [file 12967_2024_5376_MOESM1_ESM.docx]

**Gut microbiota-derived acetate attenuates lung injury induced by influenza infection via protecting airway tight junctions**

Lei Hu^1,2†^, Li Sun^1,2†^, Chun Yang^3^, Da-Wei Zhang^1,2^, Yuan-Yuan Wei^1,2^, Ming-Ming Yang^1,2^, Hui-Mei Wu^2,4^, Guang-He Fei^1,2^*

^1^ Department of Respiratory and Critical Care Medicine, First Affiliated Hospital of Anhui Medical University, Hefei, China;

^2^ Key Laboratory of Respiratory Diseases Research and Medical Transformation of Anhui Province, Hefei, China;

^3^Department of Emergency Intensive Care Unit, First Affiliated Hospital of Anhui Medical University, Hefei, China;

^4^Anhui Geriatric Institute, Department of Geriatric Respiratory and Critical Care Medicine, The First Affiliated Hospital of Anhui Medical University, Hefei, China.

**Correspondence**

Guang-He Fei E-mail address: [gh.fei@ahmu.edu.cn](mailto:gh.fei@ahmu.edu.cn)

Lei Hu and Li Sun contributed equally to this work.

**Supplemental materials and methods**

**1. Cell viability assay**

HBE cells (5×10^3^ cells/well) were cultured in 96-well plates and stimulated accordingly. After 24 h, the cells were incubated with Cell Counting Kit-8 (CCK-8, 10μl) for 2 h. The spectrometric absorbance at 450 nm was determined with a microplate reader ( Thermo Scientific, Waltham, MA, USA), and all experiments were repeated five times to ensure consistent results.


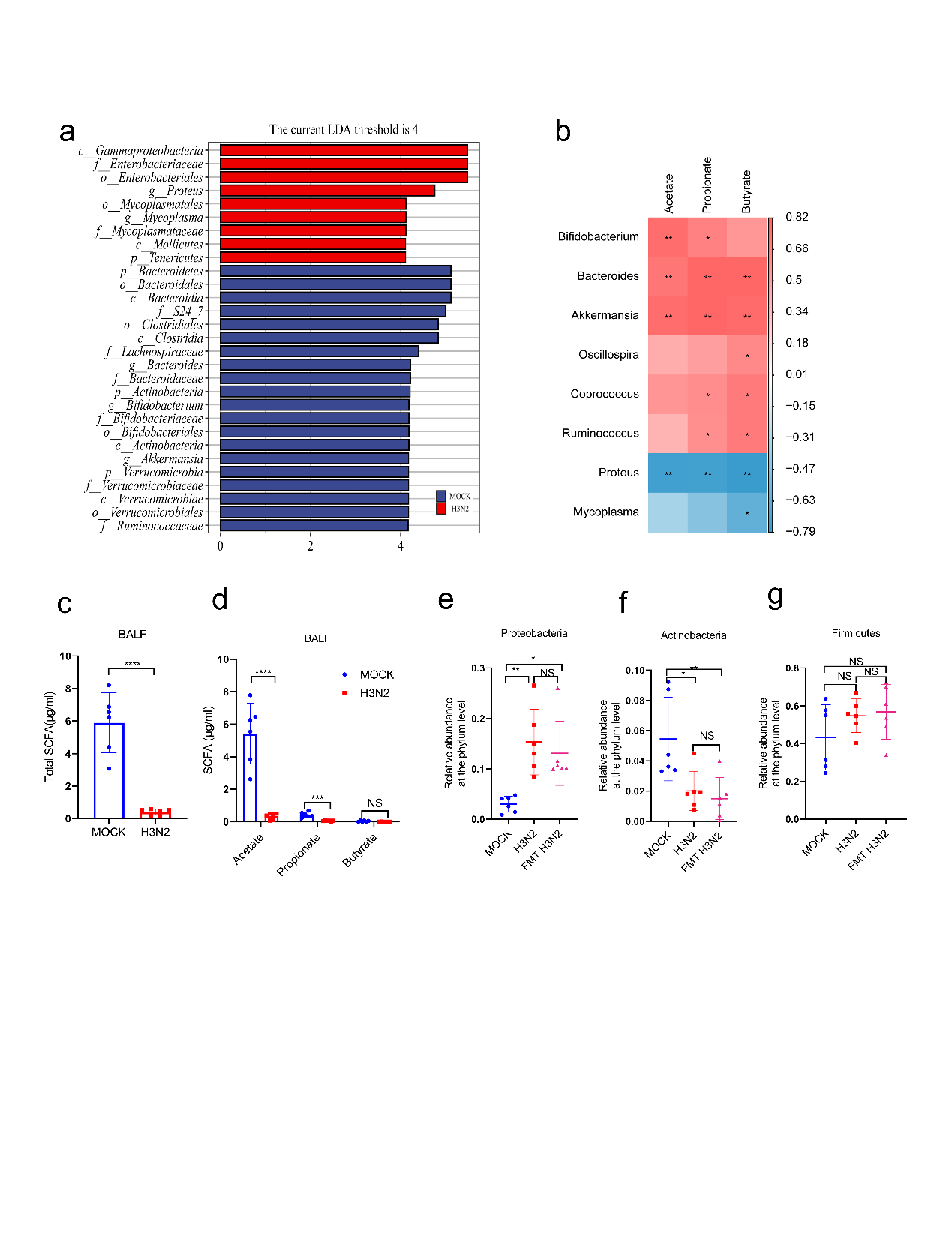


**Figure S1:** Joint analysis of fecal microbiota and SCFAs. (a) Analysis with the linear discriminant analysis (LDA). (b) Spearman correlation analysis of GM and SCFAs. (c-d) Concentrations of total SCFAs and individual concentrations of acetate, propionate, and butyrate in BALF of mice. (e-g) The relative abundances of Proteobacteria , Actinobacteria and Firmicutes in three groups were analyzed.


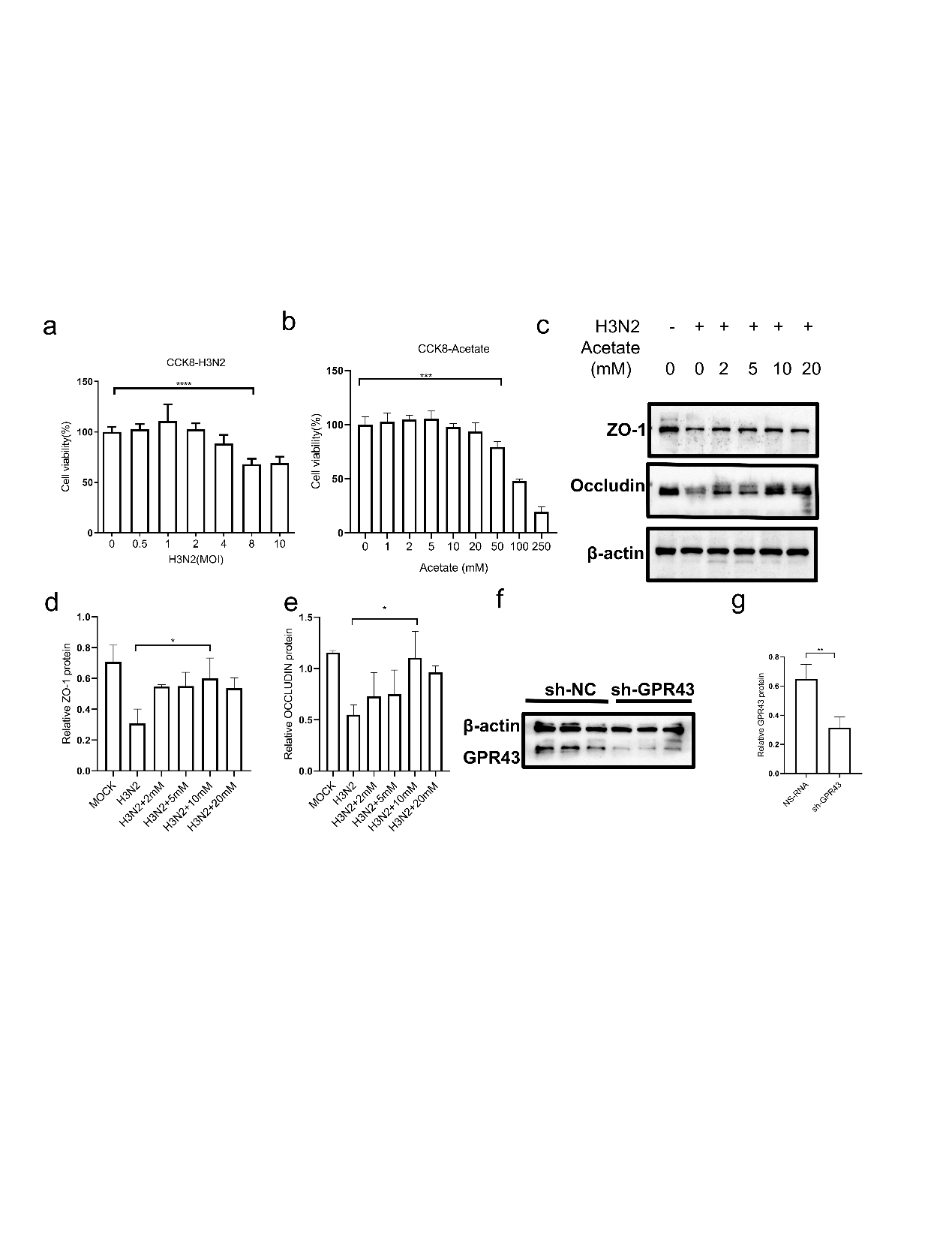


**Figure S2:** (a) Effect of H3N2 with different MOI on cell viability. (b): Effect of different concentrations of acetate on cell viability. (c-e) Analysis of occludin and ZO-1 expression by western blotting in HBE cells after H3N2 infection in the presence of different concentrations acetate. The expression of occludin and ZO-1 were quantified by Image J. (f-g) HBE cells were transfected with sh-NC or GPR43-specific shRNA. Analysis of GPR43 protein expression by western blotting.


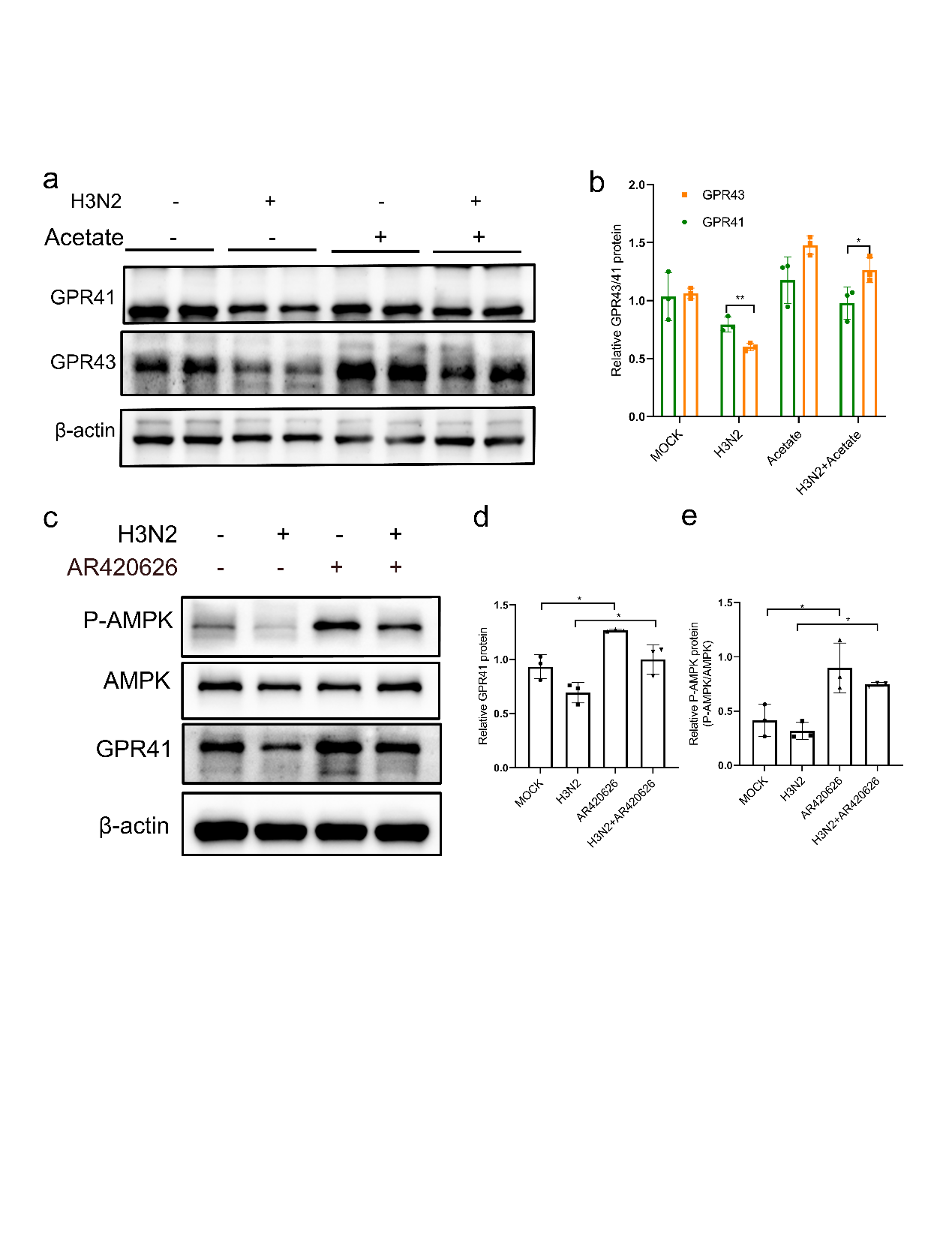


**Figure S3:** GPR41 up-regulated P-AMPK expression during IAV infection. (a-b) HBE cells were infected with H3N2 (MOI 4) for 24 h in the presence or absence of acetate (10 mM). Analysis of GPR41 and GPR43 expression by western blotting . The expression of GPR41 and GPR43 were quantified by Image J. (c-e) HBE cells were infected with H3N2 (MOI 4) for 24 h in the presence or absence of AR420626 (10 μM). Analysis of GPR41 , AMPK and P-AMPK expression by western blotting . The expression of GPR41 , AMPK and P-AMPK were quantified by Image J.


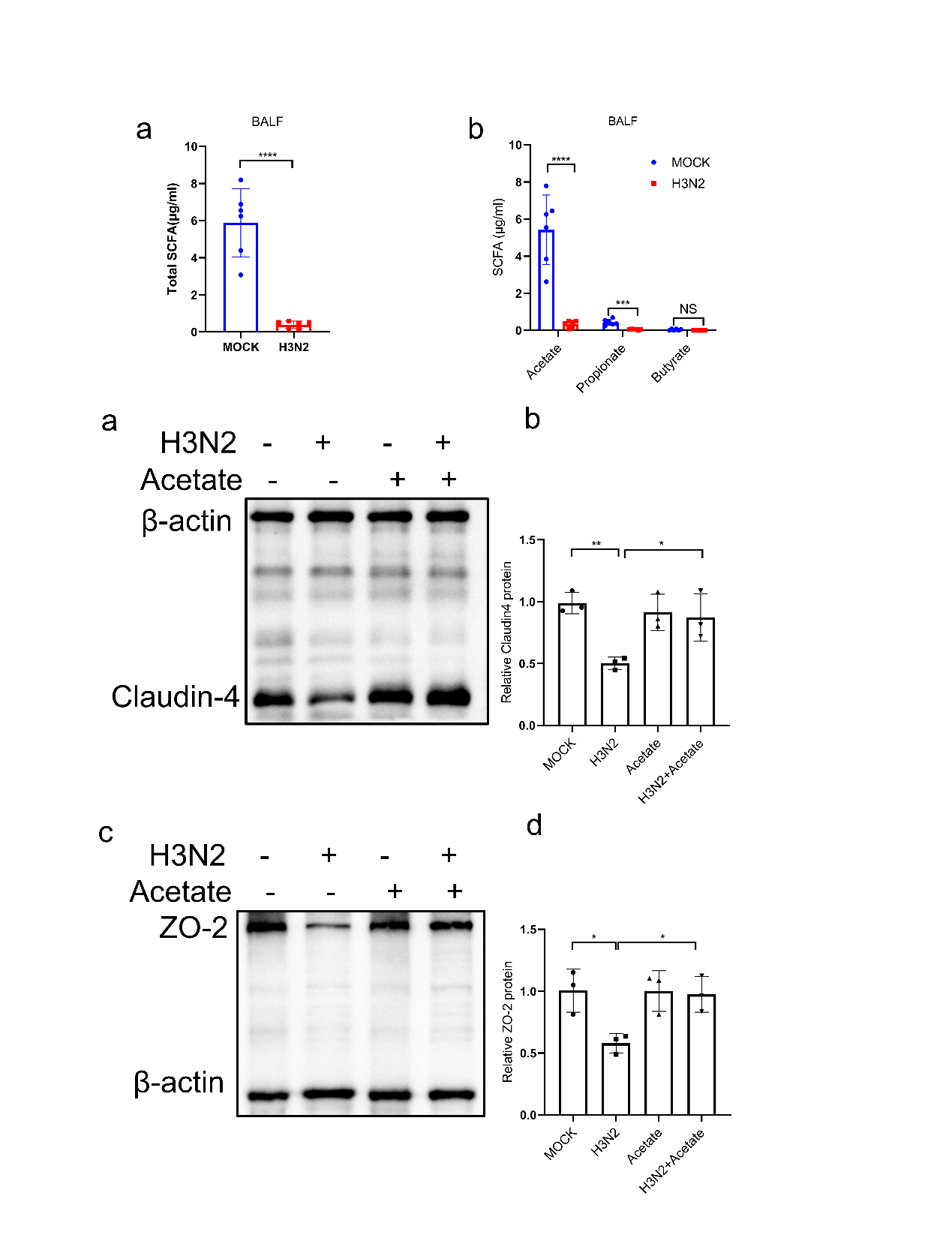


**Figure S4:** Acetate protected epithelial TJs protein. (a-d) HBE cells were infected with H3N2 (MOI 4) for 24 h in the presence or absence of acetate (10 mM). Analysis of Claudin-4 and ZO-2 expression by western blotting . The expression of Claudin-4 and ZO-2were quantified by Image J.
